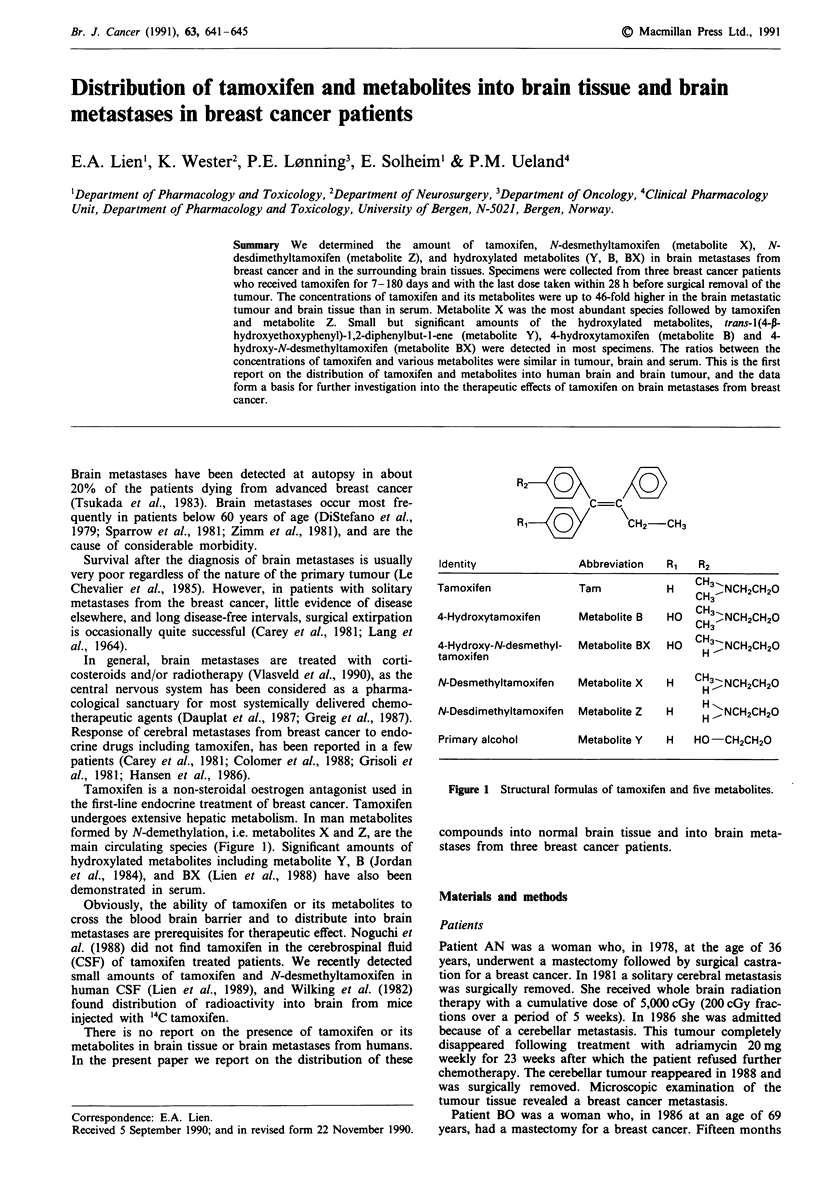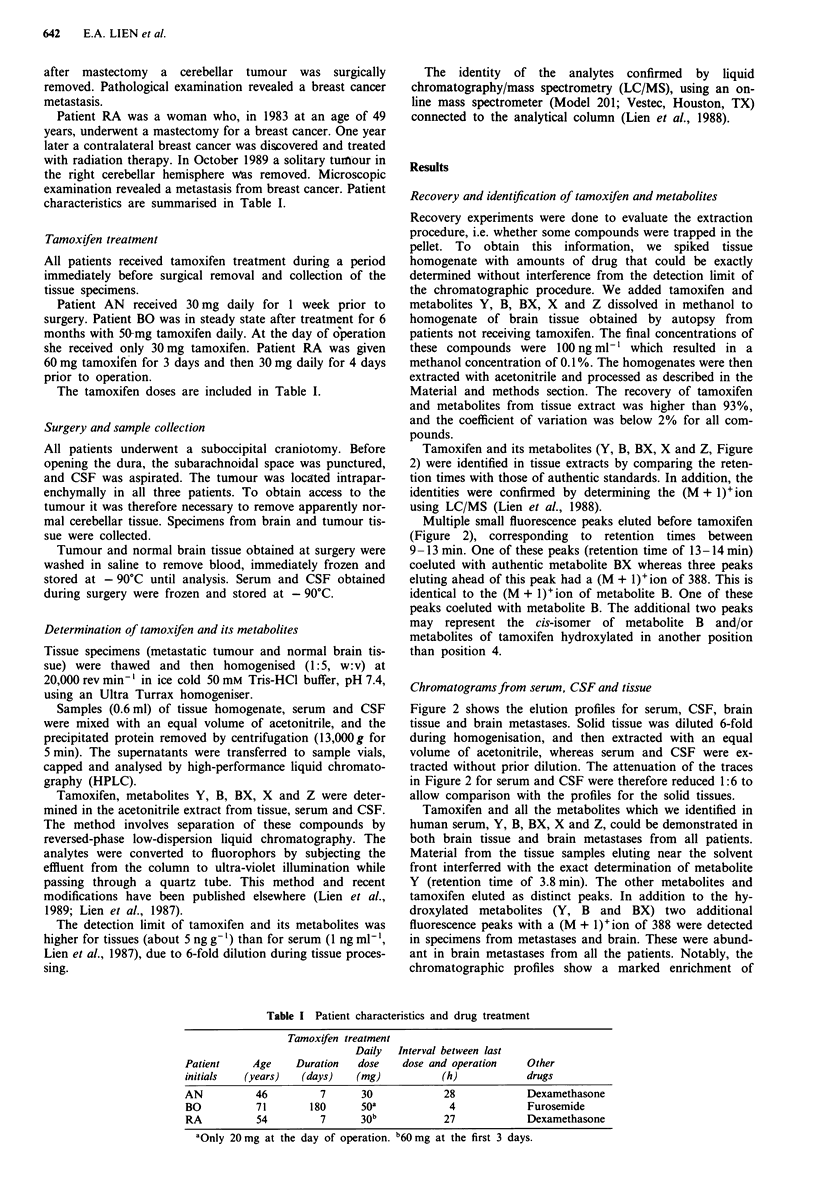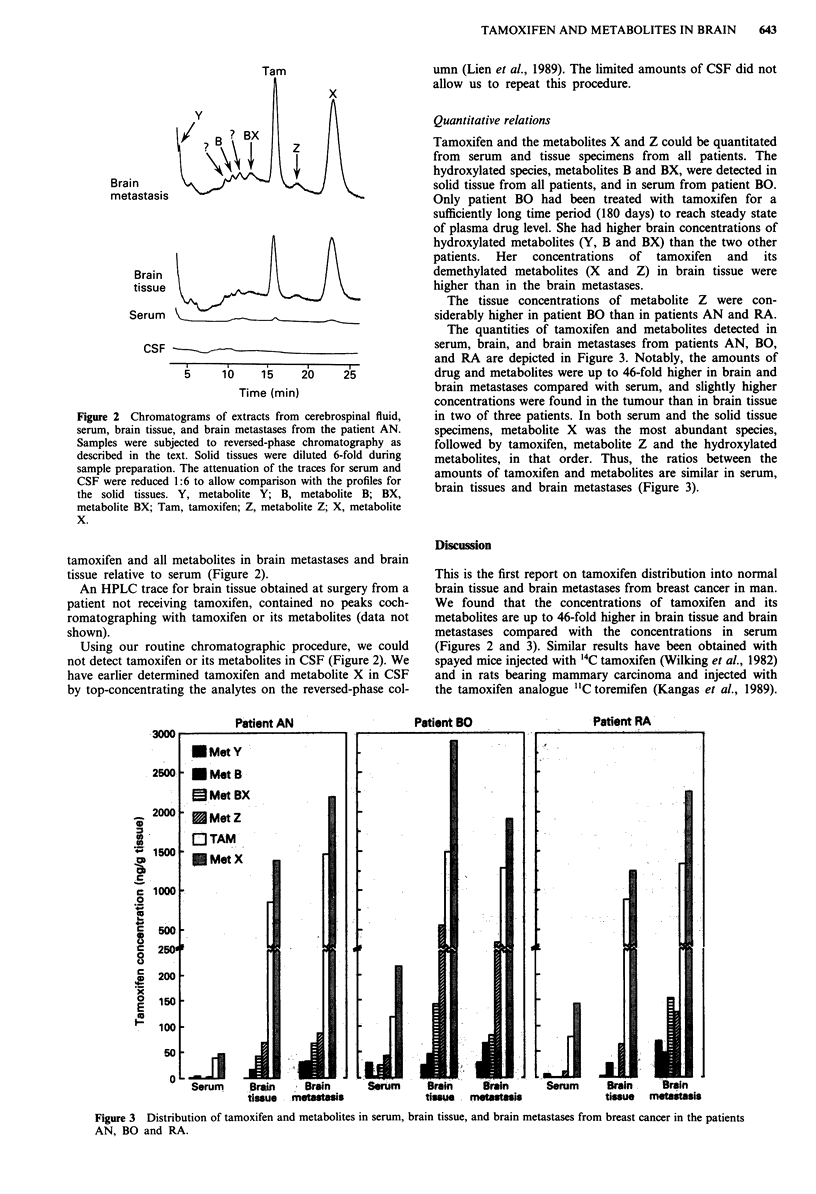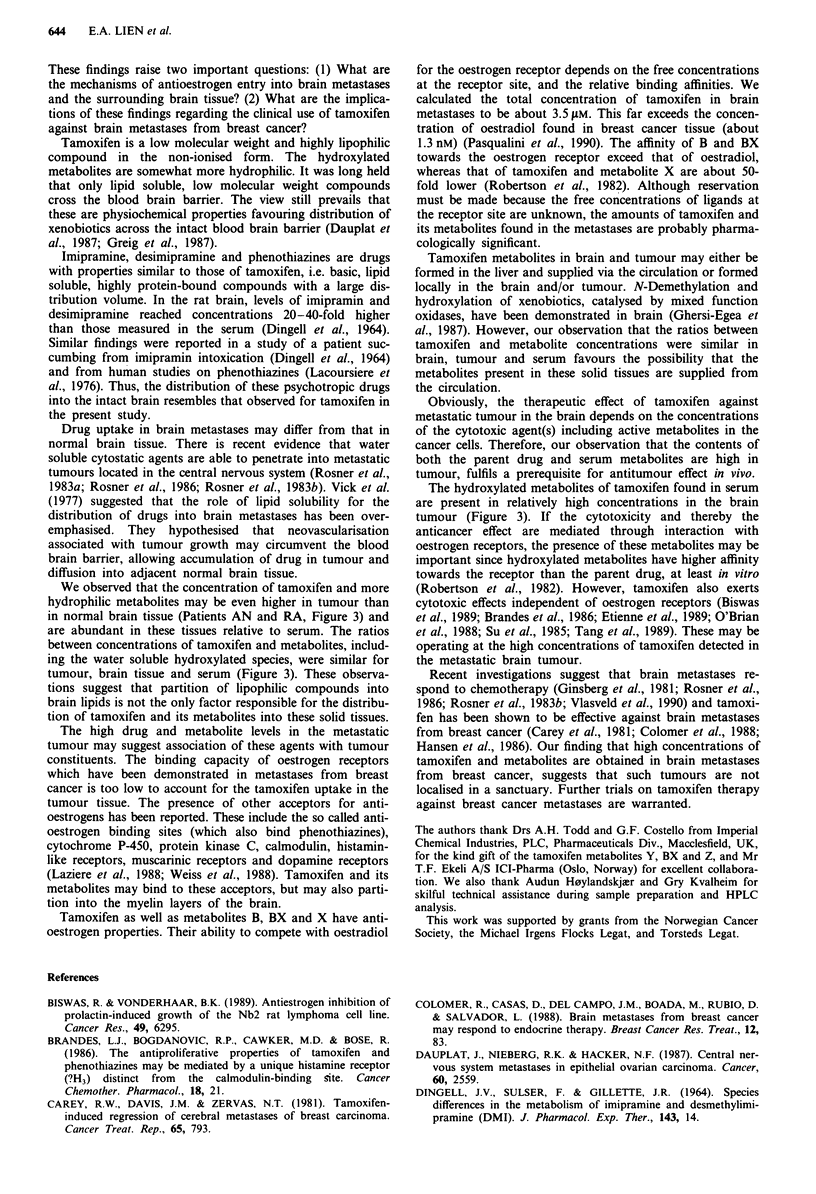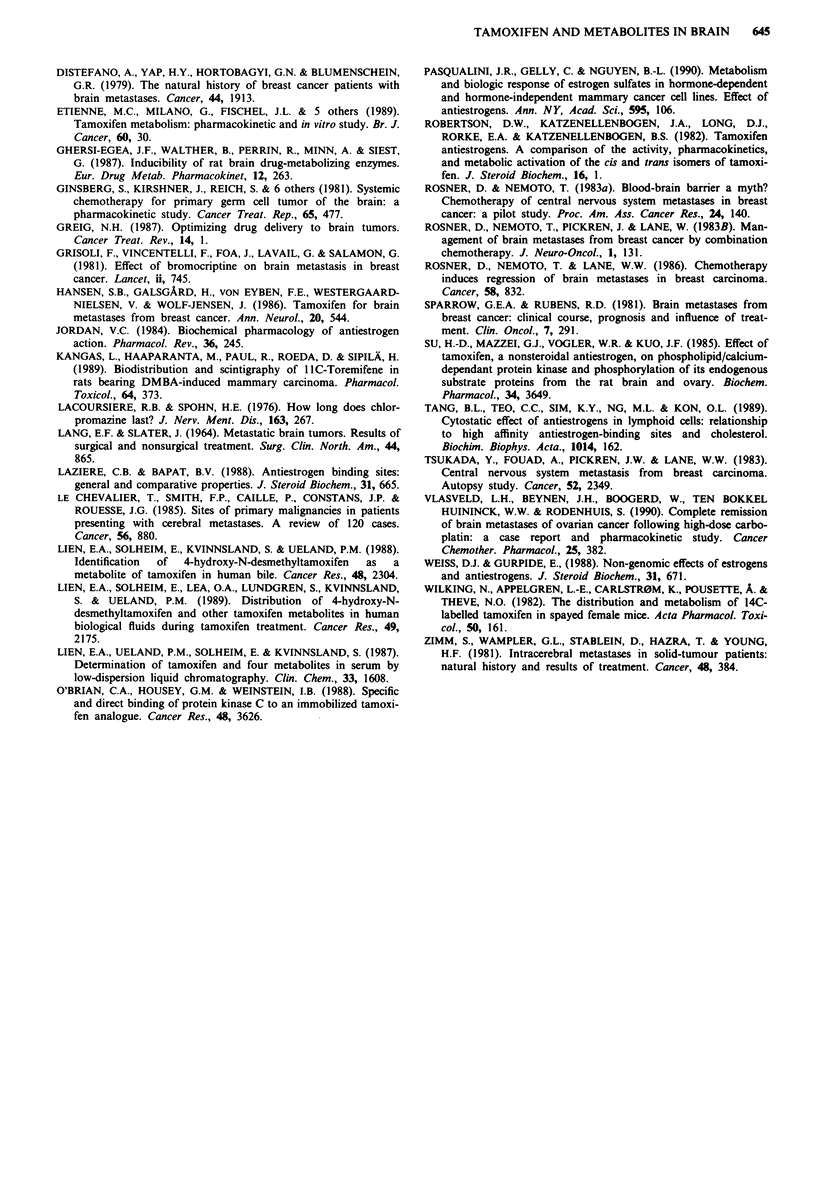# Distribution of tamoxifen and metabolites into brain tissue and brain metastases in breast cancer patients.

**DOI:** 10.1038/bjc.1991.147

**Published:** 1991-04

**Authors:** E. A. Lien, K. Wester, P. E. Lønning, E. Solheim, P. M. Ueland

**Affiliations:** Department of Pharmacology and Toxicology, University of Bergen, Norway.

## Abstract

We determined the amount of tamoxifen, N-desmethyltamoxifen (metabolite X), N-desdimethyltamoxifen (metabolite Z), and hydroxylated metabolites (Y, B, BX) in brain metastases from breast cancer and in the surrounding brain tissues. Specimens were collected from the breast cancer patients who received tamoxifen for 7-180 days and with the last dose taken within 28 h before surgical removal of the tumour. The concentrations of tamoxifen and its metabolites were up to 46-fold higher in the brain metastatic tumour and brain tissue than in serum. Metabolite X was the most abundant species followed by tamoxifen and metabolite Z. Small but significant amounts of the hydroxylated metabolites, trans-1(4-beta-hydroxyethoxyphenyl)-1,2-diphenylbut-1-ene (metabolite Y), 4-hydroxytamoxifen (metabolite B) and 4-hydroxy-N-desmethyltamoxifen (metabolite BX) were detected in most specimens. The ratios between the concentrations of tamoxifen and various metabolites were similar in tumour, brain and serum. This is the first report on the distribution of tamoxifen and metabolites into human brain and brain tumour, and the data form a basis for further investigation into the therapeutic effects of tamoxifen on brain metastases from breast cancer.


Br. J. Cancer (1991), 63, 641-645 © Macmillan Press Ltd., 1991

### Distribution of tamoxifen and metabolites into brain tissue and brain metastases in breast cancer patients

E.A. Lien[1], K. Wester[2], P.E. Lønning[3], E. Solheim[1] & P.M. Ueland[4]

[1]*Department of Pharmacology and Toxicology,* [2]*Department of Neurosurgery,* [3]*Department of Oncology,* [4]*Clinical Pharmacology Unit, Department of Pharmacology and Toxicology, University of Bergen, N-5021, Bergen, Norway.*

**Summary** We determined the amount of tamoxifen, *N*-desmethyltamoxifen (metabolite X), *N*-desdimethyltamoxifen (metabolite Z), and hydroxylated metabolites (Y, B, BX) in brain metastases from breast cancer and in the surrounding brain tissues. Specimens were collected from three breast cancer patients who received tamoxifen for 7-180 days and with the last dose taken within 28 h before surgical removal of the tumour. The concentrations of tamoxifen and its metabolites were up to 46-fold higher in the brain metastatic tumour and brain tissue than in serum. Metabolite X was the most abundant species followed by tamoxifen and metabolite Z. Small but significant amounts of the hydroxylated metabolites, *trans*-1(4-β-hydroxyethoxyphenyl)-1,2-diphenylbut-1-ene (metabolite Y), 4-hydroxytamoxifen (metabolite B) and 4-hydroxy-*N*-desmethyltamoxifen (metabolite BX) were detected in most specimens. The ratios between the concentrations of tamoxifen and various metabolites were similar in tumour, brain and serum. This is the first report on the distribution of tamoxifen and metabolites into human brain and brain tumour, and the data form a basis for further investigation into the therapeutic effects of tamoxifen on brain metastases from breast cancer.

Brain metastases have been detected at autopsy in about 20% of the patients dying from advanced breast cancer (Tsukada *et al.*, 1983). Brain metastases occur most frequently in patients below 60 years of age (DiStefano *et al.*, 1979; Sparrow *et al.*, 1981; Zimm *et al.*, 1981), and are the cause of considerable morbidity.

Survival after the diagnosis of brain metastases is usually very poor regardless of the nature of the primary tumour (Le Chevalier *et al.*, 1985). However, in patients with solitary metastases from the breast cancer, little evidence of disease elsewhere, and long disease-free intervals, surgical extirpation is occasionally quite successful (Carey *et al.*, 1981; Lang *et al.*, 1964).

In general, brain metastases are treated with corticosteroids and/or radiotherapy (Vlasveld *et al.*, 1990), as the central nervous system has been considered as a pharmacological sanctuary for most systemically delivered chemotherapeutic agents (Dauplat *et al.*, 1987; Greig *et al.*, 1987). Response of cerebral metastases from breast cancer to endocrine drugs including tamoxifen, has been reported in a few patients (Carey *et al.*, 1981; Colomer *et al.*, 1988; Grisoli *et al.*, 1981; Hansen *et al.*, 1986).

Tamoxifen is a non-steroidal oestrogen antagonist used in the first-line endocrine treatment of breast cancer. Tamoxifen undergoes extensive hepatic metabolism. In man metabolites formed by *N*-demethylation, i.e. metabolites X and Z, are the main circulating species (Figure 1). Significant amounts of hydroxylated metabolites including metabolite Y, B (Jordan *et al.*, 1984), and BX (Lien *et al.*, 1988) have also been demonstrated in serum.

Obviously, the ability of tamoxifen or its metabolites to cross the blood brain barrier and to distribute into brain metastases are prerequisites for therapeutic effect. Noguchi *et al.* (1988) did not find tamoxifen in the cerebrospinal fluid (CSF) of tamoxifen treated patients. We recently detected small amounts of tamoxifen and *N*-desmethyltamoxifen in human CSF (Lien *et al.*, 1989), and Wilking *et al.* (1982) found distribution of radioactivity into brain from mice injected with $^{14}C$ tamoxifen.

There is no report on the presence of tamoxifen or its metabolites in brain tissue or brain metastases from humans. In the present paper we report on the distribution of these compounds into normal brain tissue and into brain metastases from three breast cancer patients.

| Identity | Abbreviation | $R_1$ | $R_2$ |
|---|---|---|---|
| Tamoxifen | Tam | H | $(CH_3)(CH_3){>}NCH_2CH_2O$ |
| 4-Hydroxytamoxifen | Metabolite B | HO | $(CH_3)(CH_3){>}NCH_2CH_2O$ |
| 4-Hydroxy-*N*-desmethyl-tamoxifen | Metabolite BX | HO | $(CH_3)(H){>}NCH_2CH_2O$ |
| *N*-Desmethyltamoxifen | Metabolite X | H | $(CH_3)(H){>}NCH_2CH_2O$ |
| *N*-Desdimethyltamoxifen | Metabolite Z | H | $(H)(H){>}NCH_2CH_2O$ |
| Primary alcohol | Metabolite Y | H | $HO—CH_2CH_2O$ |

**Figure 1** Structural formulas of tamoxifen and five metabolites.

### Materials and methods

### *Patients*

Patient AN was a woman who, in 1978, at the age of 36 years, underwent a mastectomy followed by surgical castration for a breast cancer. In 1981 a solitary cerebral metastasis was surgically removed. She received whole brain radiation therapy with a cumulative dose of 5,000 cGy (200 cGy fractions over a period of 5 weeks). In 1986 she was admitted because of a cerebellar metastasis. This tumour completely disappeared following treatment with adriamycin 20 mg weekly for 23 weeks after which the patient refused further chemotherapy. The cerebellar tumour reappeared in 1988 and was surgically removed. Microscopic examination of the tumour tissue revealed a breast cancer metastasis.

Patient BO was a woman who, in 1986 at an age of 69 years, had a mastectomy for a breast cancer. Fifteen months

Correspondence: E.A. Lien.
Received 5 September 1990; and in revised form 22 November 1990.

after mastectomy a cerebellar tumour was surgically removed. Pathological examination revealed a breast cancer metastasis.

Patient RA was a woman who, in 1983 at an age of 49 years, underwent a mastectomy for a breast cancer. One year later a contralateral breast cancer was discovered and treated with radiation therapy. In October 1989 a solitary tumour in the right cerebellar hemisphere was removed. Microscopic examination revealed a metastasis from breast cancer. Patient characteristics are summarised in Table I.

*Tamoxifen treatment*

All patients received tamoxifen treatment during a period immediately before surgical removal and collection of the tissue specimens.

Patient AN received 30 mg daily for 1 week prior to surgery. Patient BO was in steady state after treatment for 6 months with 50 mg tamoxifen daily. At the day of operation she received only 30 mg tamoxifen. Patient RA was given 60 mg tamoxifen for 3 days and then 30 mg daily for 4 days prior to operation.

The tamoxifen doses are included in Table I.

*Surgery and sample collection*

All patients underwent a suboccipital craniotomy. Before opening the dura, the subarachnoidal space was punctured, and CSF was aspirated. The tumour was located intraparenchymally in all three patients. To obtain access to the tumour it was therefore necessary to remove apparently normal cerebellar tissue. Specimens from brain and tumour tissue were collected.

Tumour and normal brain tissue obtained at surgery were washed in saline to remove blood, immediately frozen and stored at − 90°C until analysis. Serum and CSF obtained during surgery were frozen and stored at − 90°C.

*Determination of tamoxifen and its metabolites*

Tissue specimens (metastatic tumour and normal brain tissue) were thawed and then homogenised (1:5, w:v) at 20,000 rev $min^{-1}$ in ice cold 50 mM Tris-HCl buffer, pH 7.4, using an Ultra Turrax homogeniser.

Samples (0.6 ml) of tissue homogenate, serum and CSF were mixed with an equal volume of acetonitrile, and the precipitated protein removed by centrifugation (13,000 *g* for 5 min). The supernatants were transferred to sample vials, capped and analysed by high-performance liquid chromatography (HPLC).

Tamoxifen, metabolites Y, B, BX, X and Z were determined in the acetonitrile extract from tissue, serum and CSF. The method involves separation of these compounds by reversed-phase low-dispersion liquid chromatography. The analytes were converted to fluorophors by subjecting the effluent from the column to ultra-violet illumination while passing through a quartz tube. This method and recent modifications have been published elsewhere (Lien *et al.*, 1989; Lien *et al.*, 1987).

The detection limit of tamoxifen and its metabolites was higher for tissues (about 5 ng $g^{-1}$) than for serum (1 ng $ml^{-1}$, Lien *et al.*, 1987), due to 6-fold dilution during tissue processing.

The identity of the analytes confirmed by liquid chromatography/mass spectrometry (LC/MS), using an online mass spectrometer (Model 201; Vestec, Houston, TX) connected to the analytical column (Lien *et al.*, 1988).

### Results

*Recovery and identification of tamoxifen and metabolites*

Recovery experiments were done to evaluate the extraction procedure, i.e. whether some compounds were trapped in the pellet. To obtain this information, we spiked tissue homogenate with amounts of drug that could be exactly determined without interference from the detection limit of the chromatographic procedure. We added tamoxifen and metabolites Y, B, BX, X and Z dissolved in methanol to homogenate of brain tissue obtained by autopsy from patients not receiving tamoxifen. The final concentrations of these compounds were 100 ng $ml^{-1}$ which resulted in a methanol concentration of 0.1%. The homogenates were then extracted with acetonitrile and processed as described in the Material and methods section. The recovery of tamoxifen and metabolites from tissue extract was higher than 93%, and the coefficient of variation was below 2% for all compounds.

Tamoxifen and its metabolites (Y, B, BX, X and Z, Figure 2) were identified in tissue extracts by comparing the retention times with those of authentic standards. In addition, the identities were confirmed by determining the $(M + 1)^+$ ion using LC/MS (Lien *et al.*, 1988).

Multiple small fluorescence peaks eluted before tamoxifen (Figure 2), corresponding to retention times between 9–13 min. One of these peaks (retention time of 13–14 min) coeluted with authentic metabolite BX whereas three peaks eluting ahead of this peak had a $(M + 1)^+$ ion of 388. This is identical to the $(M + 1)^+$ ion of metabolite B. One of these peaks coeluted with metabolite B. The additional two peaks may represent the *cis*-isomer of metabolite B and/or metabolites of tamoxifen hydroxylated in another position than position 4.

*Chromatograms from serum, CSF and tissue*

Figure 2 shows the elution profiles for serum, CSF, brain tissue and brain metastases. Solid tissue was diluted 6-fold during homogenisation, and then extracted with an equal volume of acetonitrile, whereas serum and CSF were extracted without prior dilution. The attenuation of the traces in Figure 2 for serum and CSF were therefore reduced 1:6 to allow comparison with the profiles for the solid tissues.

Tamoxifen and all the metabolites which we identified in human serum, Y, B, BX, X and Z, could be demonstrated in both brain tissue and brain metastases from all patients. Material from the tissue samples eluting near the solvent front interferred with the exact determination of metabolite Y (retention time of 3.8 min). The other metabolites and tamoxifen eluted as distinct peaks. In addition to the hydroxylated metabolites (Y, B and BX) two additional fluorescence peaks with a $(M + 1)^+$ ion of 388 were detected in specimens from metastases and brain. These were abundant in brain metastases from all the patients. Notably, the chromatographic profiles show a marked enrichment of

**Table I** Patient characteristics and drug treatment

| *Patient initials* | *Age (years)* | *Tamoxifen treatment Duration (days)* | *Daily dose (mg)* | *Interval between last dose and operation (h)* | *Other drugs* |
|---|---|---|---|---|---|
| AN | 46 | 7 | 30 | 28 | Dexamethasone |
| BO | 71 | 180 | 50[a] | 4 | Furosemide |
| RA | 54 | 7 | 30[b] | 27 | Dexamethasone |

[a]Only 20 mg at the day of operation. [b]60 mg at the first 3 days.

**Figure 2** Chromatograms of extracts from cerebrospinal fluid, serum, brain tissue, and brain metastases from the patient AN. Samples were subjected to reversed-phase chromatography as described in the text. Solid tissues were diluted 6-fold during sample preparation. The attenuation of the traces for serum and CSF were reduced 1:6 to allow comparison with the profiles for the solid tissues. Y, metabolite Y; B, metabolite B; BX, metabolite BX; Tam, tamoxifen; Z, metabolite Z; X, metabolite X.

tamoxifen and all metabolites in brain metastases and brain tissue relative to serum (Figure 2).

An HPLC trace for brain tissue obtained at surgery from a patient not receiving tamoxifen, contained no peaks cochromatographing with tamoxifen or its metabolites (data not shown).

Using our routine chromatographic procedure, we could not detect tamoxifen or its metabolites in CSF (Figure 2). We have earlier determined tamoxifen and metabolite X in CSF by top-concentrating the analytes on the reversed-phase column (Lien *et al.*, 1989). The limited amounts of CSF did not allow us to repeat this procedure.

### *Quantitative relations*

Tamoxifen and the metabolites X and Z could be quantitated from serum and tissue specimens from all patients. The hydroxylated species, metabolites B and BX, were detected in solid tissue from all patients, and in serum from patient BO. Only patient BO had been treated with tamoxifen for a sufficiently long time period (180 days) to reach steady state of plasma drug level. She had higher brain concentrations of hydroxylated metabolites (Y, B and BX) than the two other patients. Her concentrations of tamoxifen and its demethylated metabolites (X and Z) in brain tissue were higher than in the brain metastases.

The tissue concentrations of metabolite Z were considerably higher in patient BO than in patients AN and RA.

The quantities of tamoxifen and metabolites detected in serum, brain, and brain metastases from patients AN, BO, and RA are depicted in Figure 3. Notably, the amounts of drug and metabolites were up to 46-fold higher in brain and brain metastases compared with serum, and slightly higher concentrations were found in the tumour than in brain tissue in two of three patients. In both serum and the solid tissue specimens, metabolite X was the most abundant species, followed by tamoxifen, metabolite Z and the hydroxylated metabolites, in that order. Thus, the ratios between the amounts of tamoxifen and metabolites are similar in serum, brain tissues and brain metastases (Figure 3).

### Discussion

This is the first report on tamoxifen distribution into normal brain tissue and brain metastases from breast cancer in man. We found that the concentrations of tamoxifen and its metabolites are up to 46-fold higher in brain tissue and brain metastases compared with the concentrations in serum (Figures 2 and 3). Similar results have been obtained with spayed mice injected with $^{14}C$ tamoxifen (Wilking *et al.*, 1982) and in rats bearing mammary carcinoma and injected with the tamoxifen analogue $^{11}C$ toremifen (Kangas *et al.*, 1989).

**Figure 3** Distribution of tamoxifen and metabolites in serum, brain tissue, and brain metastases from breast cancer in the patients AN, BO and RA.

These findings raise two important questions: (1) What are the mechanisms of antioestrogen entry into brain metastases and the surrounding brain tissue? (2) What are the implications of these findings regarding the clinical use of tamoxifen against brain metastases from breast cancer?

Tamoxifen is a low molecular weight and highly lipophilic compound in the non-ionised form. The hydroxylated metabolites are somewhat more hydrophilic. It was long held that only lipid soluble, low molecular weight compounds cross the blood brain barrier. The view still prevails that these are physiochemical properties favouring distribution of xenobiotics across the intact blood brain barrier (Dauplat *et al.*, 1987; Greig *et al.*, 1987).

Imipramine, desimipramine and phenothiazines are drugs with properties similar to those of tamoxifen, i.e. basic, lipid soluble, highly protein-bound compounds with a large distribution volume. In the rat brain, levels of imipramin and desimipramine reached concentrations 20–40-fold higher than those measured in the serum (Dingell *et al.*, 1964). Similar findings were reported in a study of a patient succumbing from imipramin intoxication (Dingell *et al.*, 1964) and from human studies on phenothiazines (Lacoursiere *et al.*, 1976). Thus, the distribution of these psychotropic drugs into the intact brain resembles that observed for tamoxifen in the present study.

Drug uptake in brain metastases may differ from that in normal brain tissue. There is recent evidence that water soluble cytostatic agents are able to penetrate into metastatic tumours located in the central nervous system (Rosner *et al.*, 1983*a*; Rosner *et al.*, 1986; Rosner *et al.*, 1983*b*). Vick *et al.* (1977) suggested that the role of lipid solubility for the distribution of drugs into brain metastases has been overemphasised. They hypothesised that neovascularisation associated with tumour growth may circumvent the blood brain barrier, allowing accumulation of drug in tumour and diffusion into adjacent normal brain tissue.

We observed that the concentration of tamoxifen and more hydrophilic metabolites may be even higher in tumour than in normal brain tissue (Patients AN and RA, Figure 3) and are abundant in these tissues relative to serum. The ratios between concentrations of tamoxifen and metabolites, including the water soluble hydroxylated species, were similar for tumour, brain tissue and serum (Figure 3). These observations suggest that partition of lipophilic compounds into brain lipids is not the only factor responsible for the distribution of tamoxifen and its metabolites into these solid tissues.

The high drug and metabolite levels in the metastatic tumour may suggest association of these agents with tumour constituents. The binding capacity of oestrogen receptors which have been demonstrated in metastases from breast cancer is too low to account for the tamoxifen uptake in the tumour tissue. The presence of other acceptors for antioestrogens has been reported. These include the so called antioestrogen binding sites (which also bind phenothiazines), cytochrome P-450, protein kinase C, calmodulin, histamin-like receptors, muscarinic receptors and dopamine receptors (Laziere *et al.*, 1988; Weiss *et al.*, 1988). Tamoxifen and its metabolites may bind to these acceptors, but may also partition into the myelin layers of the brain.

Tamoxifen as well as metabolites B, BX and X have antioestrogen properties. Their ability to compete with oestradiol for the oestrogen receptor depends on the free concentrations at the receptor site, and the relative binding affinities. We calculated the total concentration of tamoxifen in brain metastases to be about 3.5 μM. This far exceeds the concentration of oestradiol found in breast cancer tissue (about 1.3 nM) (Pasqualini *et al.*, 1990). The affinity of B and BX towards the oestrogen receptor exceed that of oestradiol, whereas that of tamoxifen and metabolite X are about 50-fold lower (Robertson *et al.*, 1982). Although reservation must be made because the free concentrations of ligands at the receptor site are unknown, the amounts of tamoxifen and its metabolites found in the metastases are probably pharmacologically significant.

Tamoxifen metabolites in brain and tumour may either be formed in the liver and supplied via the circulation or formed locally in the brain and/or tumour. *N*-Demethylation and hydroxylation of xenobiotics, catalysed by mixed function oxidases, have been demonstrated in brain (Ghersi-Egea *et al.*, 1987). However, our observation that the ratios between tamoxifen and metabolite concentrations were similar in brain, tumour and serum favours the possibility that the metabolites present in these solid tissues are supplied from the circulation.

Obviously, the therapeutic effect of tamoxifen against metastatic tumour in the brain depends on the concentrations of the cytotoxic agent(s) including active metabolites in the cancer cells. Therefore, our observation that the contents of both the parent drug and serum metabolites are high in tumour, fulfils a prerequisite for antitumour effect *in vivo*.

The hydroxylated metabolites of tamoxifen found in serum are present in relatively high concentrations in the brain tumour (Figure 3). If the cytotoxicity and thereby the anticancer effect are mediated through interaction with oestrogen receptors, the presence of these metabolites may be important since hydroxylated metabolites have higher affinity towards the receptor than the parent drug, at least *in vitro* (Robertson *et al.*, 1982). However, tamoxifen also exerts cytotoxic effects independent of oestrogen receptors (Biswas *et al.*, 1989; Brandes *et al.*, 1986; Etienne *et al.*, 1989; O'Brian *et al.*, 1988; Su *et al.*, 1985; Tang *et al.*, 1989). These may be operating at the high concentrations of tamoxifen detected in the metastatic brain tumour.

Recent investigations suggest that brain metastases respond to chemotherapy (Ginsberg *et al.*, 1981; Rosner *et al.*, 1986; Rosner *et al.*, 1983*b*; Vlasveld *et al.*, 1990) and tamoxifen has been shown to be effective against brain metastases from breast cancer (Carey *et al.*, 1981; Colomer *et al.*, 1988; Hansen *et al.*, 1986). Our finding that high concentrations of tamoxifen and metabolites are obtained in brain metastases from breast cancer, suggests that such tumours are not localised in a sanctuary. Further trials on tamoxifen therapy against breast cancer metastases are warranted.

The authors thank Drs A.H. Todd and G.F. Costello from Imperial Chemical Industries, PLC, Pharmaceuticals Div., Macclesfield, UK, for the kind gift of the tamoxifen metabolites Y, BX and Z, and Mr T.F. Ekeli A/S ICI-Pharma (Oslo, Norway) for excellent collaboration. We also thank Audun Høylandskjær and Gry Kvalheim for skilful technical assistance during sample preparation and HPLC analysis.

This work was supported by grants from the Norwegian Cancer Society, the Michael Irgens Flocks Legat, and Torsteds Legat.

### References

BISWAS, R. & VONDERHAAR, B.K. (1989). Antiestrogen inhibition of prolactin-induced growth of the Nb2 rat lymphoma cell line. *Cancer Res.*, **49**, 6295.

BRANDES, L.J., BOGDANOVIC, R.P., CAWKER, M.D. & BOSE, R. (1986). The antiproliferative properties of tamoxifen and phenothiazines may be mediated by a unique histamine receptor (?$H_3$) distinct from the calmodulin-binding site. *Cancer Chemother. Pharmacol.*, **18**, 21.

CAREY, R.W., DAVIS, J.M. & ZERVAS, N.T. (1981). Tamoxifen-induced regression of cerebral metastases of breast carcinoma. *Cancer Treat. Rep.*, **65**, 793.

COLOMER, R., CASAS, D., DEL CAMPO, J.M., BOADA, M., RUBIO, D. & SALVADOR, L. (1988). Brain metastases from breast cancer may respond to endocrine therapy. *Breast Cancer Res. Treat.*, **12**, 83.

DAUPLAT, J., NIEBERG, R.K. & HACKER, N.F. (1987). Central nervous system metastases in epithelial ovarian carcinoma. *Cancer*, **60**, 2559.

DINGELL, J.V., SULSER, F. & GILLETTE, J.R. (1964). Species differences in the metabolism of imipramine and desmethylimipramine (DMI). *J. Pharmacol. Exp. Ther.*, **143**, 14.

DISTEFANO, A., YAP, H.Y., HORTOBAGYI, G.N. & BLUMENSCHEIN, G.R. (1979). The natural history of breast cancer patients with brain metastases. *Cancer*, **44**, 1913.

ETIENNE, M.C., MILANO, G., FISCHEL, J.L. & 5 others (1989). Tamoxifen metabolism: pharmacokinetic and *in vitro* study. *Br. J. Cancer*, **60**, 30.

GHERSI-EGEA, J.F., WALTHER, B., PERRIN, R., MINN, A. & SIEST, G. (1987). Inducibility of rat brain drug-metabolizing enzymes. *Eur. Drug Metab. Pharmacokinet*, **12**, 263.

GINSBERG, S., KIRSHNER, J., REICH, S. & 6 others (1981). Systemic chemotherapy for primary germ cell tumor of the brain: a pharmacokinetic study. *Cancer Treat. Rep.*, **65**, 477.

GREIG, N.H. (1987). Optimizing drug delivery to brain tumors. *Cancer Treat. Rev.*, **14**, 1.

GRISOLI, F., VINCENTELLI, F., FOA, J., LAVAIL, G. & SALAMON, G. (1981). Effect of bromocriptine on brain metastasis in breast cancer. *Lancet*, **ii**, 745.

HANSEN, S.B., GALSGÅRD, H., VON EYBEN, F.E., WESTERGAARD-NIELSEN, V. & WOLF-JENSEN, J. (1986). Tamoxifen for brain metastases from breast cancer. *Ann. Neurol.*, **20**, 544.

JORDAN, V.C. (1984). Biochemical pharmacology of antiestrogen action. *Pharmacol. Rev.*, **36**, 245.

KANGAS, L., HAAPARANTA, M., PAUL, R., ROEDA, D. & SIPILÄ, H. (1989). Biodistribution and scintigraphy of 11C-Toremifene in rats bearing DMBA-induced mammary carcinoma. *Pharmacol. Toxicol.*, **64**, 373.

LACOURSIERE, R.B. & SPOHN, H.E. (1976). How long does chlorpromazine last? *J. Nerv. Ment. Dis.*, **163**, 267.

LANG, E.F. & SLATER, J. (1964). Metastatic brain tumors. Results of surgical and nonsurgical treatment. *Surg. Clin. North. Am.*, **44**, 865.

LAZIERE, C.B. & BAPAT, B.V. (1988). Antiestrogen binding sites: general and comparative properties. *J. Steroid Biochem.*, **31**, 665.

LE CHEVALIER, T., SMITH, F.P., CAILLE, P., CONSTANS, J.P. & ROUESSE, J.G. (1985). Sites of primary malignancies in patients presenting with cerebral metastases. A review of 120 cases. *Cancer*, **56**, 880.

LIEN, E.A., SOLHEIM, E., KVINNSLAND, S. & UELAND, P.M. (1988). Identification of 4-hydroxy-N-desmethyltamoxifen as a metabolite of tamoxifen in human bile. *Cancer Res.*, **48**, 2304.

LIEN, E.A., SOLHEIM, E., LEA, O.A., LUNDGREN, S., KVINNSLAND, S. & UELAND, P.M. (1989). Distribution of 4-hydroxy-N-desmethyltamoxifen and other tamoxifen metabolites in human biological fluids during tamoxifen treatment. *Cancer Res.*, **49**, 2175.

LIEN, E.A., UELAND, P.M., SOLHEIM, E. & KVINNSLAND, S. (1987). Determination of tamoxifen and four metabolites in serum by low-dispersion liquid chromatography. *Clin. Chem.*, **33**, 1608.

O'BRIAN, C.A., HOUSEY, G.M. & WEINSTEIN, I.B. (1988). Specific and direct binding of protein kinase C to an immobilized tamoxifen analogue. *Cancer Res.*, **48**, 3626.

PASQUALINI, J.R., GELLY, C. & NGUYEN, B.-L. (1990). Metabolism and biologic response of estrogen sulfates in hormone-dependent and hormone-independent mammary cancer cell lines. Effect of antiestrogens. *Ann. NY, Acad. Sci.*, **595**, 106.

ROBERTSON, D.W., KATZENELLENBOGEN, J.A., LONG, D.J., RORKE, E.A. & KATZENELLENBOGEN, B.S. (1982). Tamoxifen antiestrogens. A comparison of the activity, pharmacokinetics, and metabolic activation of the *cis* and *trans* isomers of tamoxifen. *J. Steroid Biochem.*, **16**, 1.

ROSNER, D. & NEMOTO, T. (1983*a*). Blood-brain barrier a myth? Chemotherapy of central nervous system metastases in breast cancer: a pilot study. *Proc. Am. Ass. Cancer Res.*, **24**, 140.

ROSNER, D., NEMOTO, T., PICKREN, J. & LANE, W. (1983*B*). Management of brain metastases from breast cancer by combination chemotherapy. *J. Neuro-Oncol.*, **1**, 131.

ROSNER, D., NEMOTO, T. & LANE, W.W. (1986). Chemotherapy induces regression of brain metastases in breast carcinoma. *Cancer*, **58**, 832.

SPARROW, G.E.A. & RUBENS, R.D. (1981). Brain metastases from breast cancer: clinical course, prognosis and influence of treatment. *Clin. Oncol.*, **7**, 291.

SU, H.-D., MAZZEI, G.J., VOGLER, W.R. & KUO, J.F. (1985). Effect of tamoxifen, a nonsteroidal antiestrogen, on phospholipid/calcium-dependant protein kinase and phosphorylation of its endogenous substrate proteins from the rat brain and ovary. *Biochem. Pharmacol.*, **34**, 3649.

TANG, B.L., TEO, C.C., SIM, K.Y., NG, M.L. & KON, O.L. (1989). Cytostatic effect of antiestrogens in lymphoid cells: relationship to high affinity antiestrogen-binding sites and cholesterol. *Biochim. Biophys. Acta.*, **1014**, 162.

TSUKADA, Y., FOUAD, A., PICKREN, J.W. & LANE, W.W. (1983). Central nervous system metastasis from breast carcinoma. Autopsy study. *Cancer*, **52**, 2349.

VLASVELD, L.H., BEYNEN, J.H., BOOGERD, W., TEN BOKKEL HUININCK, W.W. & RODENHUIS, S. (1990). Complete remission of brain metastases of ovarian cancer following high-dose carboplatin: a case report and pharmacokinetic study. *Cancer Chemother. Pharmacol.*, **25**, 382.

WEISS, D.J. & GURPIDE, E., (1988). Non-genomic effects of estrogens and antiestrogens. *J. Steroid Biochem.*, **31**, 671.

WILKING, N., APPELGREN, L.-E., CARLSTRØM, K., POUSETTE, Å. & THEVE, N.O. (1982). The distribution and metabolism of 14C-labelled tamoxifen in spayed female mice. *Acta Pharmacol. Toxicol.*, **50**, 161.

ZIMM, S., WAMPLER, G.L., STABLEIN, D., HAZRA, T. & YOUNG, H.F. (1981). Intracerebral metastases in solid-tumour patients: natural history and results of treatment. *Cancer*, **48**, 384.